# Supplementary material for: Identification of photoperiod-induced specific miRNAs in the adrenal glands of Sunite sheep (Ovis aries)
Source: Front Vet Sci. 2022 Jul 22;9:888207. doi: 10.3389/fvets.2022.888207 (PMC9354845; doi:10.3389/fvets.2022.888207)
Supplement: Supplementary file 1 [file Data_Sheet_1.ZIP › Supplementary file/miRDeep2 file.zip!/miRDeep2 file/Novel_Structure/SPLP42A2/Novel_404.pdf]

Provisional ID	:	NC_019473.2_30491
Score total	:	1.2
Score for star read(s)	:	-1.3
Score for read counts	:	-0.3
Score for mfe	:	1.3
Score for randfold	:	1.6
Score for cons. seed	:	
Total read count	:	11
Mature read count	:	11
Loop read count	:	0
Star read count	:	0

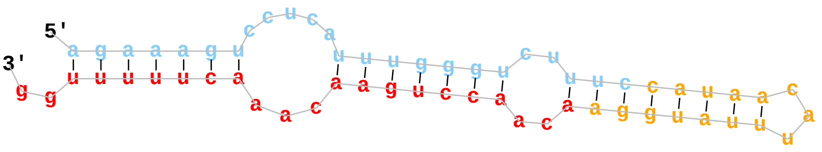

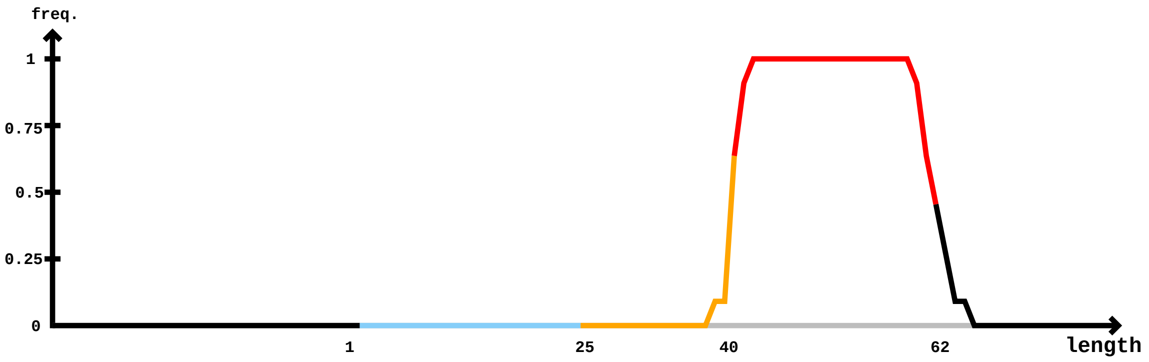


```
                                    Star                                            Mature
5'- aaauacaacaggaaaagguauggguugacuagaaaguccucauuugggucuuuccauaacauuuauggaacaaccugaacaaacuuuuuggccaacccaagaacucuua   -3'    exp
    ..............(((((.(((((((.(((((((((.....(((((((..((((((((...))))))))..)))))))...))))))))).)))))))...)).))).      reads       mm          sample
    ...................................................................gaaAaaccugaacaaacuuuu.....................      1           1           seq
    .....................................................................aAaaccugaacaaacuuuuu....................      2           1           seq
    .....................................................................aAaaccugaacaaacuuuuug...................      2           1           seq
    .....................................................................aAaaccugaacaaacuuuuugg..................      2           1           seq
    ......................................................................caaccugaacaaacuuuuu....................      1           0           seq
    ......................................................................caaccugaacaaacuuuuuggc.................      1           0           seq
    ......................................................................Gaaccugaacaaacuuuuuggc.................      1           1           seq
    .......................................................................aaccugaacGaacuuuuuggcca...............      1           1           seq
```
